# Efficacy of Levocetirizine in Reducing Double‐J Stent–Related Symptoms: A Double‐Blind Pilot Randomized Controlled Trial

**DOI:** 10.1155/aiu/9326991

**Published:** 2026-07-22

**Authors:** Marc Elias, Rami Halaby, Anthony Mina, Ziad Azar, Serge Assaf, Ghida El-Khoury, Maryline Ghosh, Sabine Breidy, Charbel Hachem, Charbel Dabal, Antoine Kassis, Raghid El-Khoury, Rodrigue Saad

**Affiliations:** ^1^ School of Medicine and Medical Sciences, Holy Spirit University of Kaslik (USEK), Jounieh, Lebanon, usek.edu.lb; ^2^ Department of Urology, Notre-Dame des Secours University Hospital, Byblos, Lebanon

**Keywords:** antihistamines, double-J stent, levocetirizine, ureteral stent, ureteroscopy, urolithiasis

## Abstract

**Purpose:**

The aim of this study is to evaluate the safety and efficacy of levocetirizine in reducing ureteral stent–related symptoms.

**Methods:**

In this single‐center randomized controlled trial, 85 patients requiring double‐J stent placement, 3 patients were excluded and 82 were allocated to start preoperatively levocetirizine 5 mg daily (Group B *n* = 41) or placebo (Group A *n* = 41) from stent insertion until stone removal. Primary outcomes were assessed using the Ureteral Stent Symptom Questionnaire (USSQ) and visual analog scale (VAS) for pain. Secondary outcomes included macroscopic assessment of ureteral inflammation and stent calcification during ureteroscopy for stone removal within 20 days.

**Results:**

Out of 82 participants included, three patients were excluded before analysis due to loss to follow‐up or hospitalized for UTI that mimics stent‐related symptoms. 79 patients were considered for final analysis. Group B showed a statistically significant reduction in stent‐related morbidity USSQ across all subgroups compared to the placebo group and a lower VAS pain score (*p* < 0.05). Macroscopic assessment during stent removal also demonstrated statistically significantly less ureteral inflammation in Group B (17.07%) vs. 82.93% in Group A (*p* < 0.005).

**Conclusion:**

This pilot study indicates that levocetirizine effectively reduces stent‐related symptoms and maintains a good safety profile. These findings suggest that levocetirizine may be a well‐tolerated adjunct therapy for managing stent discomfort.

**Trial registration:** Clinical trial.gov.identifier: LBCTR2025075754

## 1. Introduction

Double‐J (DJ) ureteral stents are widely used in urological practice to maintain ureteral drainage after procedures such as ureteroscopy or in cases of obstructive uropathy [1, 2]. However, their placement is often associated with discomfort and bothersome and lower urinary tract symptoms, including dysuria, urinary urgency, frequency, hematuria, and flank pain [3]. These symptoms can significantly impair patients’ quality of life (QoL) as assessed by validated tools like the Ureteral Stent Symptom Questionnaire (USSQ) [4].

The pathophysiology of pain and discomfort related to DJ stents is still not fully understood. Several studies have suggested that mechanical irritation of the bladder trigone and ureter, rather than purely chemical irritation, contributes to symptoms [5]. Despite modifications to stent design such as the development of single‐J or loop‐tail configurations, symptoms persist in many patients, suggesting that mechanisms beyond physical irritation may be involved [6].

Clinical experience reveals considerable variability in individual tolerance to ureteral stents. Some patients remain largely asymptomatic, while others report significant discomfort that interferes with daily activities [4]. Notably, patients with long‐term indwelling stents placed for neoplastic obstruction and maintained under immunosuppressive chemotherapy often tolerate stents remarkably well. This observation raises the possibility that an immune‐mediated or allergic response may contribute to symptom variability.

Histamine, released by mast cells, is known to promote bladder inflammation, visceral hypersensitivity, and ureteral smooth muscle contractions [7, 8]. By inhibiting mast cell activation and modulating eosinophil activity, antihistamines have demonstrated efficacy in reducing symptoms associated with bladder pain syndrome and acute renal colic [8, 9]. Preliminary clinical observations also indicate that longer indwelling times of ureteral stents correlate with more severe eosinophilic inflammation, reinforcing he hypothesis that local allergic‐type reactions may be involved [10].

In vitro studies have demonstrated that histamine can directly increase ureteral contractility by stimulating smooth muscle cells and enhancing peristaltic activity [11], yet the potential utility of antihistamines for stent‐related symptoms remains underexplored.

Cetirizine and its active enantiomer levocetirizine are second‐generation H1‐receptor antagonists with favorable safety and minimal sedative effects [12]. Importantly, levocetirizine is predominantly excreted unchanged in urine [13], suggesting that it may achieve high concentrations within the urinary tract and potentially mitigate local irritative mechanisms.

Given the high prevalence of DJ stent placement in our daily practice and the limited effectiveness of current management strategies, novel therapeutic approaches are needed.

This pilot randomized controlled trial aims to evaluate the efficacy and safety of levocetirizine (XYZAL) compared to placebo in reducing DJ stent–related symptoms and improving QoL in adult patients undergoing ureteral stenting for obstructive disease.

## 2. Materials and Methods

### 2.1. Trial Design, Approval, and Registration

**FIGURE 1:**
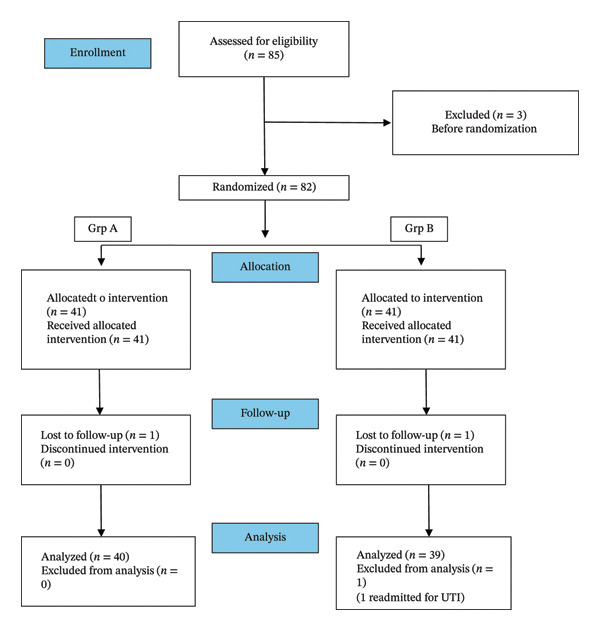
CONSORT flowchart for the study participants. CONSORT flow diagram illustrating the enrollment, allocation, follow‐up, and analysis of participants in the randomized controlled trial evaluating levocetirizine for double‐J stent–related symptoms. The diagram details the number of patients assessed for eligibility, randomly assigned to each study group, received the allocated intervention, lost to follow‐up, and included in the final analysis.

This single‐center, pilot, randomized, double‐blind, placebo‐controlled trial was conducted at CHU–Notre Dame des Secours University Hospital, Byblos, Lebanon, between March 2025 and October 2025. The study was approved by the Institutional Review Board of CHU–NDS University Hospital (Approval No. CR:3/2024) and registered in the Lebanese Clinical Trials Registry (LBCTR2025075754) on 3 March 2025, retrospectively registered. Written informed consent was obtained from all participants. The study was conducted and reported in accordance with the Consolidated Standards of Reporting Trials (CONSORT) guidelines. The CONSORT flowchart illustrates the recruitment and allocation process (Figure 1).

### 2.2. Participants

The trial included Lebanese adults aged 18–80 years old undergoing DJ stent placement for obstructive ureteral disease under the care of 5 urologists. Exclusion criteria consisted of: known allergies or contraindications to the study drug; a history of radiation‐induced ureteritis, malignancy, or extrinsic ureteral obstruction; previous ureteral stenting; pregnancy or breastfeeding; end‐stage renal disease (glomerular filtration rate < 10 mL/min) or on hemodialysis; solitary or transplanted kidney; and refusal to provide written informed consent. It should be noted that 3 patients were excluded prior to randomization, one patient on hemodialysis and 2 patients were already taking antihistamines or corticosteroids in the previous month, and a further 2 patients were excluded after randomization due to loss to follow up (*n* = 2), and 1 patient was excluded from analysis since he was readmitted for UTI, since their symptoms could influence their self‐reported USSQ responses.

### 2.3. Randomization and Blinding

Eligible patients who met the inclusion criteria (*n* = 82) were randomized in a 1:1 ratio using the sealed opaque envelope technique with a block size of four to ensure allocation concealment and balance within clinics. Each prepared block contained two assignments for Group A and two for Group B. Participants were sequentially asked to select an envelope, which was then removed from the pool for the next selection within that block. This process guaranteed that for every four consecutive participants, two were assigned to each study group, ensuring balanced group sizes at baseline.

The trial was conducted as a double‐blind study. Both participants and investigators were blinded to treatment allocation. Levocetirizine and placebo tablets were identical packaging. They were prepared and dispensed by an independent party not involved in patient care, data collection, or outcome assessment. Blinding was maintained throughout the study period and was only broken after completion of data analysis.

### 2.4. Intervention

After obtaining signed informed consent, 82 participants who met the inclusion criteria were allocated into two groups. Group A (*n* = 41) received 1 tablet of placebo once daily starting at least 2 h preoperatively with the standard analgesic and anti‐inflammatory treatment post DJ stent placement (maximum of 20 days). Group B (*n* = 41) received 1 tablet of levocetirizine 5 mg (XYZAL, UCB Pharma, Brussels, Belgium) once daily starting at least 2 h preoperatively with the standard analgesic and anti‐inflammatory treatment post DJ stent placement (max. 20 days).

### 2.5. Study Drugs

Levocetirizine dihydrochloride is a potent second‐generation selective H1‐receptor antagonist approved by the U.S. Food and Drug Administration. It was selected for this trial based on its well‐established efficacy, favorable safety profile, and distinctive pharmacokinetic properties. Clinical studies have consistently demonstrated its effectiveness in reducing inflammatory and allergic symptoms with a low incidence of adverse effects [14, 15]. Approximately 85% of an administered dose is excreted unchanged in the urine via glomerular filtration and active tubular secretion [15], ensuring direct exposure of the ureteral mucosa and indwelling stent to therapeutically relevant drug concentrations. The minimal penetration of the blood–brain barrier reduces the risk of sedative and anticholinergic effects associated with first‐generation agents [16, 17]. The once‐daily 5 mg dosing regimen is well tolerated and promotes high patient adherence, thereby minimizing variability in drug exposure and enhancing the reliability of outcome assessments in this study.

In addition to the study drug (levocetirizine), all patients received standard postoperative analgesic therapy. This included paracetamol 1000 mg every 6 h as needed and ketoprofen 100 mg every 12 h as needed. Narcotic analgesia was not routinely prescribed at discharge and was reserved for breakthrough pain only. Alpha‐1 receptor antagonists were permitted postoperatively at the discretion of the operating surgeon. Patients who were already on alpha‐1 receptor antagonists prior to the study were allowed to continue their regimen. Their use was documented and later analyzed to assess potential imbalance between study groups.

### 2.6. Outcome

The primary study outcome was assessed on the day of ureteroscopy, prior to stent removal and stone extraction. Upon admission, 10‐cm visual analog scale (VAS) was completed to all patients for pain evaluation ranging from 0 (no pain) to 10 (worst imaginable pain) and the USSQ to quantify stent‐related symptoms experienced during the entire indwelling period. During the subsequent ureteroscopy procedure, the operating surgeon evaluated three macroscopic parameters under direct vision: the presence or absence of stent encrustation or calcification, inflammation of the ureteral orifice (characterized by edema or erythema), and macroscopic inflammation of the ureteral mucosa (assessed for edema, erythema, or mucosal swelling). These evaluations were conducted while patients remained under the effect of the study medication—Group A receiving daily placebo and Group B receiving 5 mg levocetirizine daily—which was initiated at least 2 hours prior to initial stent placement and discontinued immediately following the ureteroscopic procedure.

### 2.7. Data Collection

Data were collected at two points using a structured in‐person questionnaire. At the time of stent placement, baseline demographic and clinical information was obtained, including age, body mass index (BMI), educational level, habits (caffeine consumption, alcohol use, and smoking status), medical and surgical history, stone characteristics (its size in millimeters and exact location in the proximal, middle, or distal ureter), and stent specifications including manufacturer, length, diameter, and stent type. The ureteral stents used in this study included Percuflex (Boston Scientific, Marlborough, MA, USA), Wellead Double‐J Ureteral Stents (Wellead Medical Co., Ltd., Guangzhou, China), Marflow Ureteral Stents (Marflow AG, Switzerland), BioTeq Double Pigtail Ureteral Stents (BioTeq Pty Ltd., South Africa), and Rocamed Ureteral Stents (Rocamed SAM, Monaco).

At the time of stent removal, prior to ureteroscopy, patients completed the VAS for pain and the USSQ to evaluate treatment efficacy, safety, and impact on QoL during the indwelling period. Simultaneously, the operating surgeon documented intraoperative macroscopic findings, including stent calcification and ureteral inflammation. This dual‐timepoint approach ensured comprehensive assessment of both baseline characteristics and outcome measures while patients were still under the effect of the study medication.

### 2.8. USSQ

It is a validated scale assessing stent‐related morbidity across six domains: urinary symptoms (11 questions), body pain (6 questions), general health (5 questions), work performance (7 questions), sexual matters (4 questions), and global QoL (1 question). Each question is scored on a Likert scale from 0 (never/not present) to 5 (always/very severe). Higher cumulative scores indicate greater symptom severity. In this study, the USSQ was administered in person or by phone at designated timepoints by a research coordinator to objectively evaluate treatment efficacy and stent‐related QoL impact.

### 2.9. VAS

It is a validated self‐reported scale consisting of a 10‐cm horizontal line anchored at “no pain” (0 cm) and “worst imaginable pain” (10 cm). Patients independently marked the line to indicate their current pain intensity, with the score quantified by measuring the distance from the zero point. Higher scores reflected greater pain severity. This method provided an objective, patient‐centered measure of stent‐related discomfort while minimizing assessor bias.

### 2.10. Statistical Analysis

Statistical analyses were performed using SPSS Version 29 (IBM Corp., Armonk, NY, USA) for data cleaning and analysis. A margin of error of 5% was applied, and a *p* value < 0.05 was considered statistically significant.

Descriptive results are presented as means ± standard deviations for continuous variables and as absolute frequencies (*N*) and percentages (%) for categorical variables.

For bivariate analysis, the normality of continuous variables was assessed using the Shapiro–Wilk test. When the distribution was normal, comparisons between two groups were conducted using Student′s *t*‐test; otherwise, the Mann–Whitney *U* test was applied.

For categorical variables, comparisons were performed using the chi‐square (*χ* ^2^) test or Fisher’s exact test when appropriate.

The mean USSQ scores were compared across different JJ stent brands using the Kruskal–Wallis test, following a significant result on the test for equality of variances. In addition, comparisons of the mean VAS scores were performed using a one‐way ANOVA, since the test for equality of variances was not significant. The study reporting follows the CONSORT 2010 statement for randomized controlled trials.

As this study was designed as a pilot randomized controlled trial, no formal a priori sample size calculation was performed. The sample size was selected to provide preliminary estimates of treatment effect, safety, and feasibility and to inform the design of future adequately powered randomized controlled trials. This approach is consistent with recommendations regarding the role of pilot studies in clinical research [18].

## 3. Results

### 3.1. CONSORT Flowchart

Out of the 85 patients initially enrolled, 79 were included in the final analysis. Three patients were excluded before randomization, one due to hemodialysis and two because they had taken antihistamines or corticosteroids in the previous month. After randomization, two patients were lost to follow‐up, and one patient from Group B was excluded before analyzing because they were readmitted for a urinary tract infection, which could have affected their responses (Figure 1).

### 3.2. Characteristics of the Population

The overall characteristics of the population are presented in Table 1. Out of the 79 analyzed patients, there were 40 patients in the placebo group (Group A) and 39 patients in the levocetirizine group (Group B). There were no statistically significant differences between study groups regarding age, sex, marital status, education, lifestyle habits, and BMI. Also, there were no statistically significant differences between the two study groups regarding baseline symptoms, stone location, ureteral stent manufacturer, stent length, or stent diameter.

**TABLE 1:** Patient characteristics and study outcomes according to treatment group.

Bivariate analysis					
Variables		Groups Mean ± SD; *N* (%)		*p* value	Test
		*A* (*N* = 40)	*B* (*N* = 39)		
Age		52.45 ± 15.88	51.95 ± 14.27	0.83	*t*‐test
Height (cm)		172.62 ± 10.67	171.38 ± 7.81	0.55	*t*‐test
Weight (kg)		87.1 ± 20.53	81.7 ± 12.30	0.15	*t*‐test
BMI		28.88 ± 4.56	28.02 ± 5.40	0.27	Mann–Whitney
VAS		6.00 ± 1.91	2.82 ± 2.54	< 0.05	Mann–Whitney
USSQ	Urinary symptoms (/55)	30.52 ± 7.03	17.87 ± 7.05	< 0.05	*t*‐test
	Pain (/40)	25.42 ± 6.14	16.97 ± 7.05	< 0.05	Mann–Whitney
	General health (/30)	20.17 ± 5.20	10.89 ± 4.84	< 0.05	Mann–Whitney
	Work performance (/35)	19.55 ± 4.69	13.38 ± 5.18	< 0.05	Mann–Whitney
	Sexual matters (/15)	7.97 ± 2.42	6.41 ± 2.76	< 0.05	Mann–Whitney
	Additional problems (/5)	2.72 ± 1.24	2.25 ± 1.31	0.006	Mann–Whitney
	Total (/180)	106.37 ± 20.37	67.79 ± 22.53	< 0.05	*t*‐test

Sex	Male	31 (53.44)	27 (46.56)	0.405	X2
	Female	9 (42.85)	12 (57.15)		

Marital status	Single	7 (46.66)	8 (53.34)	0.73	X2
	Married	33 (51.57)	31 (48.43)		

	Primary	14 (53.84)	12 (46.16)	0.82	X2
Education status	Secondary	8 (44.44)	10 (55.56)		
	University	18 (51.43)	17 (48.57)		

Tobacco	Yes	25 (54.35)	21 (45.65)	0.43	X2
	No	15 (45.45)	18 (54.54)		

Caffeine	Yes	36 (51.43)	34 (48.57)	0.69	X2
	No	4 (44.44)	5 (55.56)		

Alcohol	Yes	28 (50.90)	27 (49.1)	0.94	X2
	No	12 (50)	12 (50)		

Symptoms	Left flank pain	23 (65.71)	12 (34.29)	0.16	X2
	Right flank pain	17 (38.64)	27 (61.36		

Alpha‐1 receptor antagonist use	Patient already using alpha antagonist (yes)	3 (7.5)	2 (5.1)	> 0.99	*t*‐test
	No	37 (92.5)	37 (94.9)		

Uroscanner	Proximal	14 (45.16)	17 (54.84)	0.58	X2
	Middle	8 (44.44)	10 (55.56)		
	Distal	12 (57.14)	9 (42.86)		
	Pelvis	6 (66.67)	3 (33.33)		

Brand	Percuflex	7 (50)	7 (50)	0.26	
	Wellead	23 (56.09)	18 (43.91)		
	Marflow	1 (20)	4 (80)		
	Biotech	7 (63.64)	4 (36.36)		
	Rocamed	2 (25)	6 (75)		

Diameter (Fr)	6	37 (51.39)	35 (48.61)	0.66	X2
	7	3 (42.86)	4 (57.14)		

Size (cm)	26 cm	8 (34.78)	15 (65.22)	0.071	X2
	28 cm	32 (57.14)	24 (42.86)		

Stent state	Calcified	25 (65.79)	13 (34.21)	0.0094	X2
	Not calcified	15 (36.59)	26 (63.41)		

Ureter state	Inflamed	34 (82.93)	7 (17.07)	< 0.005	X2
	Not inflamed	6 (15.79)	32 (84.21)		

*Note:* Data are presented as mean ± standard deviation or number (percentage). *p* values in bold indicate statistical significance (*p* < 0.05). Group A: placebo; Group B: levocetirizine 5 mg once daily.

Abbreviations: USSQ = Ureteral Stent Symptom Questionnaire; VAS = visual analog scale.

Similarly, the use of alpha‐1 receptor antagonists was uncommon and comparable between groups (7.5% in Group A versus 5.1% in Group B; Fisher’s exact test, *p* > 0.99).

### 3.3. Primary Outcomes: Symptom Scores and Macroscopic Findings

The results of the bivariate analysis comparing the primary outcomes between the levocetirizine (Group B) and placebo (Group A) groups are presented in Table 1.

### 3.4. Ureteral Stent–Related Symptoms (USSQ Questionnaire)

The main results are summarized in Table 1. Patients who took levocetirizine reported significantly less severe stent‐related symptoms across all domains of the USSQ. The total USSQ score was significantly lower in Group B (67.79 ± 22.53) compared to Group A (106.37 ± 20.37, *p* < 0.05). Significant improvements were also noted in the urinary symptoms (*p* < 0.05), body pain (*p* < 0.05), general health (*p* < 0.05), work performance (*p* < 0.05), sexual matters (*p* < 0.05), and additional problems (*p* = 0.006) subdomains.

### 3.5. Pain Assessment (VAS Scale)

**FIGURE 2:**
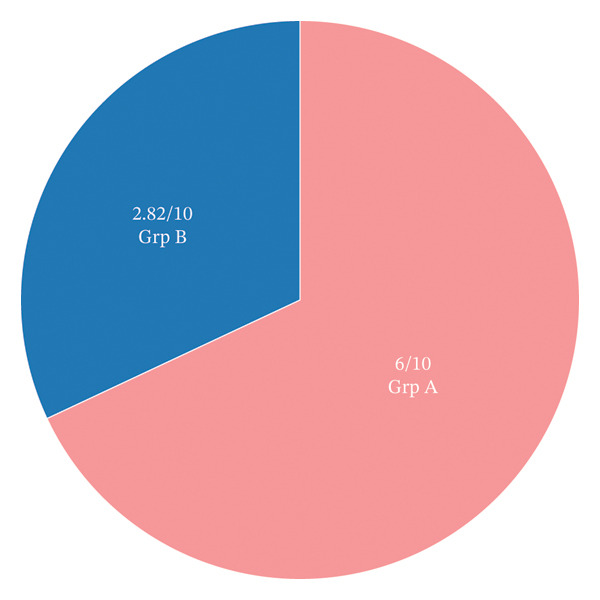
Comparison of postoperative pain intensity assessed by the visual analog scale (VAS) between the two study groups. The mean VAS pain score was significantly lower in the levocetirizine group (Group B) compared with the placebo group (Group A). Patients in Group B reported a mean VAS score of 2.82 ± 2.54, whereas those in Group A had a mean score of 6.00 ± 1.91. This difference was statistically significant (*p* < 0.05).

The mean pain score on the VAS was significantly lower in the levocetirizine group (Group B) (2.82 ± 2.54) than in the placebo group (Group A) (6.00 ± 1.91, *p* < 0.05) (Figure 2).

### 3.6. Macroscopic Intraoperative Findings

**FIGURE 3:**
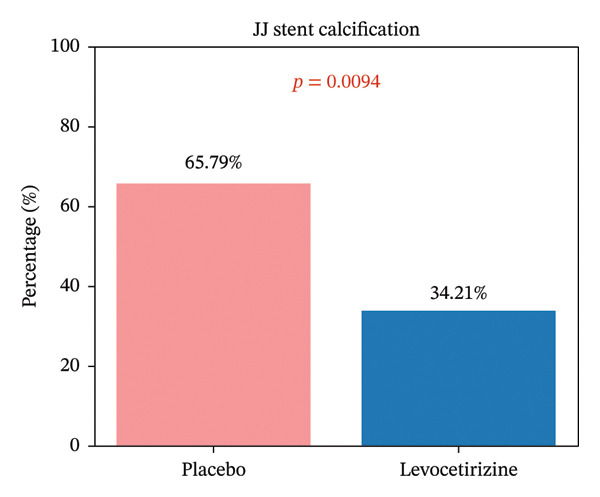
Frequency of stent calcification observed during ureteroscopy in the two study groups. Objective ureteroscopy assessment revealed a significantly lower rate of stent calcification in patients receiving levocetirizine (Group B) compared with those receiving placebo (Group A). Stent calcification was present in 34.21% of patients in Group B versus 65.79% in Group A (*p* = 0.0094).

**FIGURE 4:**
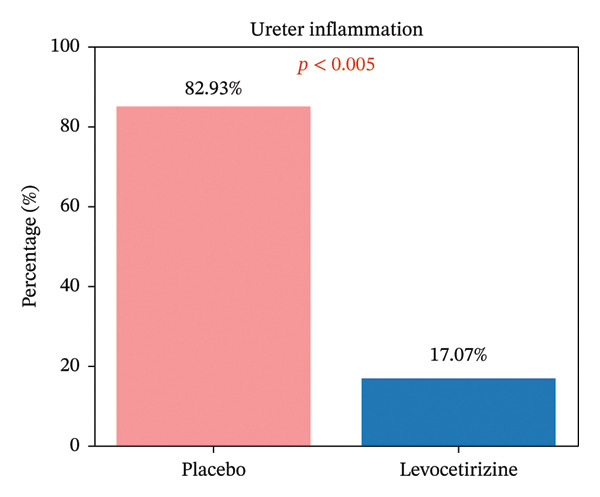
Prevalence of ureteral inflammation assessed during ureteroscopy in the levocetirizine and placebo groups. Ureteroscopy evaluation demonstrated a significantly reduced prevalence of ureteral inflammation in the levocetirizine group (Group B) compared with the placebo group (Group A). An inflamed ureter was identified in 17.07% of patients receiving levocetirizine versus 82.93% of those receiving placebo (*p* < 0.005).

Objective assessment during ureteroscopy revealed statistically significant differences between the groups. Stent calcification was less frequent in Group B (34.21% vs. 65.79%, *p* = 0.0094) (Figure 3). Also, the prevalence of an inflamed ureter was significantly lower in patients receiving levocetirizine (17.07%) compared to those receiving placebo (82.93%, *p* < 0.005) (Figure 4).

### 3.7. Safety and Tolerability

Levocetirizine was well tolerated, with a safety profile comparable to that of the placebo. There were no documented serious adverse effects, such as those requiring hospitalization, resulting in permanent disability, or requiring therapy discontinuation reported in either group. It is noteworthy that one patient was readmitted for a urinary tract infection; however, this event was not related to the study drug.

## 4. Discussion

### 4.1. Principal Findings

This pilot randomized controlled trial evaluated the safety and efficacy of levocetirizine in reducing ureteral stent–related symptoms. Patients receiving 5 mg of levocetirizine daily experienced a significant reduction in stent‐associated morbidity and pain compared with those receiving placebo, as measured by the USSQ [4], the VAS, and intraoperative assessments of ureteral inflammation. Levocetirizine showed a safety profile comparable to placebo, underscoring its potential as a well‐tolerated adjunctive therapy for managing stent‐related discomfort.

### 4.2. Interpretation

DJ ureteral stents represent a persistent clinical paradox: while they effectively relieve urinary obstruction, they are also a major source of patient morbidity [1]. Despite numerous published studies, optimal management of stent‐related discomfort remains a significant challenge in daily urological practice often providing incomplete relief accompanied by significant morbidity, including pain, urinary urgency, frequency, and decreased QoL [3]. The pathophysiology of these symptoms is increasingly recognized as multifactorial [2, 5]. While mechanical irritation is a key component, the persistence of symptoms despite stent design modification, such as single‐J or loop‐tail configurations, suggests the involvement of other mechanisms [5]. However, the considerable variability in individual symptom tolerance and the observed tolerance in immunosuppressed patients support the hypothesis that an immune‐mediated or allergic‐type reaction to the foreign body may be a contributing factor. Histamine, a key mediator in such reactions against foreign body (DJ), is known to promote inflammation, visceral hypersensitivity, and smooth muscle contractions in the urinary tract. This biological rationale also formed the basis for evaluating ureteral inflammation and stent calcification as secondary outcomes. We hypothesized that inhibition of histamine‐mediated inflammatory pathways could reduce local tissue inflammation induced by the indwelling stent. The significantly lower prevalence of ureteral inflammation observed in the levocetirizine group supports this hypothesis. Similarly, because inflammatory responses contribute to biofilm formation and early stent encrustation, attenuation of local inflammation may partly explain the lower rate of stent calcification observed in patients receiving levocetirizine. However, these findings should be considered exploratory and require confirmation through dedicated mechanistic studies. This provides a compelling rationale for exploring antihistamines as a novel therapeutic strategy for stent‐related discomfort [7, 8].

A randomized, double‐blind controlled trial by Han et al., published in Urology Practice, the official journal of the American Urological Association (AUA), evaluated the efficacy of antihistamine therapy in reducing ureteral stent‐related symptoms. [19]. This research was a pioneering effort to test this hypothesis. Building upon their work, our study incorporates several important methodological refinements to more accurately evaluate the therapeutic potential of antihistamines.

First, the choice of antihistamine is critical. While Han et al. used fexofenadine, we selected levocetirizine, a second‐generation H1‐receptor antagonist with a distinct pharmacokinetic profile that is ideally suited for urological applications. The crucial distinction lies in their routes of elimination: approximately 85% of a levocetirizine dose is excreted unchanged in the urine, ensuring direct and potent exposure of the ureteral mucosa and indwelling stent to therapeutically relevant drug concentrations. In contrast, fexofenadine is predominantly excreted in the feces, with less than 5% recovered in the urine. This fundamental difference in renal elimination likely results in significantly higher local drug levels at the critical stent–urothelium interface with levocetirizine, providing a more potent and targeted effect against the histamine‐mediated component of stent‐related inflammation [13, 15].

Our clinical findings built upon earlier laboratory work by Borgstedt et al. published as early as 1962 that proved direct in vitro evidence for this mechanism, demonstrating that histamine acts as a potent stimulant of canine ureteral smooth muscle, provoking dose‐dependent contractions. Critically, this excitatory effect was shown to be specifically mediated by histamine receptors, as it could be abolished by H1‐receptor antagonists. This establishes a direct pathophysiological link: the foreign body effect of a DJ stent likely incites local histamine release, which in turn triggers ureteral spasms and bladder trigone irritation, culminating in the characteristic symptoms of pain, urgency, and frequency. The symptom relief observed in our trial is therefore achieved by blocking these same H1‐receptors, directly countering the mechanism identified in foundational laboratory studies [20].

Second, the timing and duration of therapy are paramount. In the Han et al. study, antihistamine administration began after ureteroscopy and stent placement and was limited to a fixed 10‐day course, despite some patients having longer stent indwelling times. This design risks allowing the inflammatory cascade to be established before intervention. In contrast, our protocol initiated levocetirizine at least 2 h preoperatively. This proactive approach aimed to achieve therapeutic blood and urinary levels before the mechanical trauma of stent insertion, potentially preventing the initiation of the allergic‐inflammatory response from the outset. Furthermore, our patients received the study drug for the entire duration of stent indwelling, ensuring continuous coverage [15].

Third, our outcome assessment incorporated an additional objective measure. While both studies utilized the validated USSQ, which relies on subjective patient reporting [4], we supplemented this with direct intraoperative macroscopic evaluation by the surgeon. Assessing the ureteral mucosa for signs of inflammation (edema and erythema) at the time of stent removal provided an objective biological correlate to the patient‐reported symptomatic improvement, strengthening the validity of our findings.

In conclusion, while the study by Han et al. provided the initial proof of concept, our trial sought to optimize the intervention by using the most renally excreted antihistamine, administering it prophylactically for the full stent duration, and incorporating an objective measure of ureteral inflammation. Our positive results suggest that this refined approach may more effectively target the underlying inflammatory pathway and establish levocetirizine as a valuable, well‐tolerated adjunct for managing the pervasive problem of stent‐related symptoms.

Preliminary findings of the present study were previously presented as a conference poster at the European Association of Urology (EAU) Annual Congress 2026 and published as a conference abstract in the *European Urology Open Science* congress supplement [21]. The present manuscript represents the complete original report of the study, including the full methodology, statistical analyses, and comprehensive results (Eur Urol Open Sci. 2026; 83 (Suppl 1):S224).

### 4.3. Limitations and Biases

First, this study was designed as a pilot randomized controlled trial, and no formal a priori sample size calculation was performed. Consequently, the relatively small sample size limits statistical power, increases the risk of Type II error, and may affect the generalizability of the findings. Therefore, the results should be considered exploratory and hypothesis‐generating and require confirmation in larger multicenter randomized controlled trials [18].

Second, the single‐center design may restrict the external validity of the findings. Third, although validated instruments such as the USSQ and VAS were used, outcomes relied partly on patient‐reported measures, which may introduce reporting bias. Larger, multicenter randomized controlled trials are warranted to confirm these findings.

In addition, alpha‐1 receptor antagonists were permitted according to surgeon preference and continued in patients already receiving them before enrollment. Although their use was infrequent and similarly distributed between study groups, residual confounding related to concomitant medical therapy cannot be completely excluded.

Additionally, ureteral inflammation and stent calcification were exploratory outcomes, and the present study was not specifically powered to evaluate these endpoints. Furthermore, these findings were based on macroscopic intraoperative assessment rather than histopathological or biochemical evaluation and should therefore be interpreted cautiously.

## 5. Conclusion

In conclusion, this pilot randomized controlled trial demonstrates that levocetirizine effectively reduces ureteral stent–related morbidity and pain, showed by significant improvements in both patient‐reported outcomes (USSQ and VAS) and objective macroscopic findings during ureteroscopy for stone ablation. Starting the treatment early, before the stent is placed, and continuing it throughout the stenting period seems to be an important reason for its success. Along with its good safety profile, these results establish levocetirizine as a well‐tolerated and promising adjunctive therapy for managing a common clinical challenge of stent‐related discomfort. The potential benefit of this study requires further investigation through larger, multicenter, double‐blind randomized controlled trials to validate these results and define its role in clinical practice.

## Funding

The authors received no financial support for the research, authorship, and/or publication of this article.

## Disclosure

This work was previously presented as a poster at the EAU Congress London 2026. Only the abstract/poster version was published in the congress materials.

## Ethics Statement

This study was approved by the Institutional Review Board of CHU Notre Dame des Secours University Hospital, Byblos, Lebanon (Approval No. CR: 3/2024). The study was conducted in accordance with the ethical standards of the institutional research committee and with the 1964 Declaration of Helsinki and its later amendments.

## Consent

Written informed consent was obtained from all individual participants included in this study prior to enrollment.

## Conflicts of Interest

The authors declare no conflicts of interest.

## Data Availability

The data that support the findings of this study are available from the corresponding authors upon reasonable request.
